# Surgical Treatment of an Infected Nonunion of the Middle Third of the Femur Associated with Femoral Shortening in a Hemophilia Patient

**DOI:** 10.1155/2016/3045262

**Published:** 2016-03-17

**Authors:** Ahmet Salduz, Özcan Kaya, Halil İbrahim Balci, Turgut Akgul, Fatih Dikici, Bülent Zülfikar, Mehmet Kocaoğlu

**Affiliations:** ^1^Istanbul Faculty of Medicine, Department of Orthopedics and Traumatology, Istanbul University, 34093 Istanbul, Turkey; ^2^Atakent Hospital, Acibadem University, Istanbul, Turkey; ^3^Oncology Institute, Division of Pediatric Hematology-Oncology, Istanbul University, Istanbul, Turkey; ^4^Memorial Şişli Hospital, Istanbul, Turkey

## Abstract

The management of nonunion and limb length discrepancy has remained a constant challenge in hemophilic patients. In this study, we aimed to present the treatment of femur infected nonunion and limb length discrepancy in a twenty-seven-year-old patient with hemophilia type A. A 27-year-old male patient with hemophilia type A referred to our institution for the treatment of right femur infected nonunion and 10 cm shortness of the femur. Resection of the nonunion site and bone-to-bone fixation with autologous bone grafting were performed. Compression to the pseudoarthrosis site and distraction from new osteotomy site were applied with the unilateral external fixator. Union was achieved, and 6 cm lengthening was obtained according to the initial length. Patient was followed up for 7 years. After this treatment, the patient is able to walk with full weight bearing on the affected extremity with 4 cm shortening which is compensated by the heel lift. The results of this case indicate that limb lengthening and treatment of nonunion with the external fixation could be reliable and effective method for hemophilic patients.

## 1. Introduction

Hemophilia A, manifesting with hemorrhage, is X-linked disorder caused by the deficiency of factors VIII. Spontaneous bleeding into joints and muscles is a feature of severe hemophilia. In orthopedic practice, presentation of these patients varies with the severity of the disease. Over the last 40 years, hemophilia treatment has changed dramatically. In particular after the introduction of factor concentrate, we come across bleeding complications and long-term complications less frequently.

The management of infected nonunion of bone remained a constant challenge in hemophilic patients as well as normal patients. The associated bone defect, shortening, deformity, and infection complicate the management. A monolateral external fixator may minimize some of the problems frequently encountered in these patients. We could not find any report or study in the literature, which mentions distraction osteogenesis in hemophiliac patients. This is the first report, which describes distraction osteogenesis for the treatment of femur infected nonunion and limb length discrepancy in a twenty-seven-year-old patient with hemophilia type A.

## 2. Case Report

A 27-year-old male patient with hemophilia type A referred to our institution for the treatment of right femur nonunion and right limb length discrepancy. He was diagnosed with hemophilia A at the age of five after he had been evaluated for a cerebral bleeding in his country. He had a right femur fracture when he was 17 years old. He was treated with casting for three years. After the removal of the cast, he was able to walk without knee flexion; one year later, he had been operated on because of the osteomyelitis at the fracture site.

**Figure 1:**
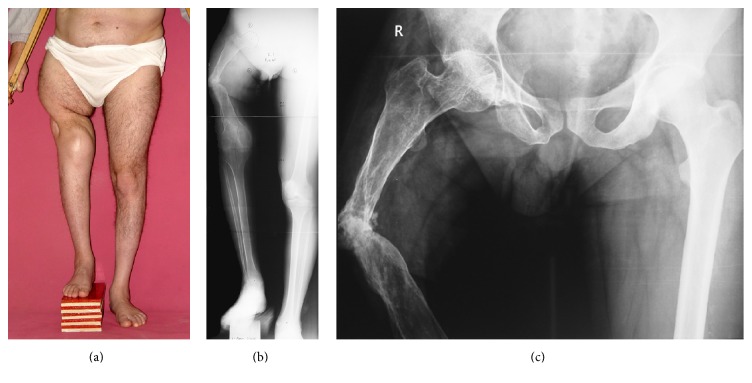
(a) Preoperative clinic appearance with 10 cm length block under the right foot. (b, c) Preoperative radiographs. Note the nonunion and osteoporosis of the right femur.

His right lower limb shortness was 10 cm and there was pathological movements at the middle of the femur (Figure 1). Old incision scar of prior surgeries was on the anterior and lateral sides of the thigh. He has left knee flexion contracture due to hemophilic arthropathy and ankylosed right knee.

Preoperative laboratory test revealed hemoglobin level of 14.8 g/dL, white blood count of 8.7 × 10^3^ *μ*L, platelets count of 259 × 10^3^ *μ*L, international normalized ratio (INR) of 1.1, and APTT of 66.4 sec (normal range: 19.5–29.1 sec). Factor VIII activity was 0% without any inhibiting factors. We consulted the patient with our Hematology Department before surgery. Preoperative, perioperative, and postoperative factor VIII and antifibrinolytic medication (tranexamic acid) protocols were given by the hematology specialist. One day before the surgery, tranexamic acid, (500 mg tablets), was started orally, four times a day. This medication was continuously given for ten days. In addition, 100 mg/mL of tranexamic acid in 100 cc isotonic fluid was given intraoperatively. Factor VIII 40 IU/kg was applied at two hours before surgery and 10 IU/kg intraoperatively.

**Figure 2:**
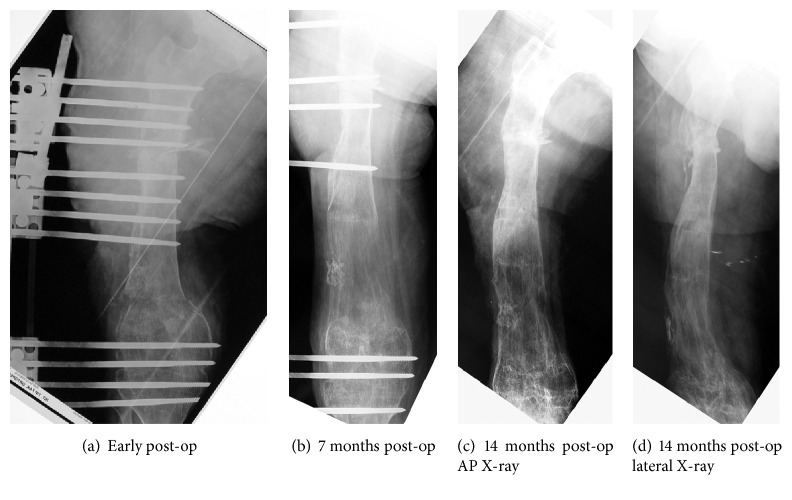
(a) Postoperative radiography of the femur. (b) Post-op 7-month radiography of the femur. (c, d) Post-op 14-month AP and lateral radiography.

The surgery was performed under general anesthesia. The patient was prepared for surgery on a radiolucent table; and the sterilization of surgical extremity was done in the standard fashion. A 5 cm incision was made just below the anterior iliac wing for harvesting bone graft. After exposing the iliac wing, periosteum of the inner table was harvested with autologous cortical and cancellous bone grafts. The incision was closed properly, after bleeding control. According the monolateral external fixator application principles, four Schanz screws were inserted into the subtrochanteric region; four Schanz screws were under the pseudoarthrosis site; four Schanz screws were below the planned second osteotomy line for lengthening perpendicular to mechanic axis; and the last two Schanz screws were inserted below the knee joint which was already ankylosed in order to increase stability by increasing the lever arm (Figure 2(a)). Previous lateral incision was used to expose pseudoarthrosis region. The segment of nonunion was removed. Unilateral external fixator was applied to the Schanz screws and then healthy live bone ends were put together to contact each other. Autologous bone grafts, which are harvested from iliac bone, were placed to the bone contact area. Periosteum, harvested from inner side of the iliac bone, was wrapped to the bone grafts and contact area. The second osteotomy was performed at the femur supracondylar region. All surgical incision was closed properly.

During the operation day, five units totally of red blood cell transfusion were needed and the control results were Hb: 11.4 mg/dL, Hct: 33.6%. After four hours from the surgery factor VIII 500 IU 8 × 1 had been started intravenously according to our local hemostasis protocol for major surgeries. Mechanical compression to bone ends was started at the nonunion site. In the postoperative second day, the patient was stabilized, but hemorrhagic drainage was inspected from the incision with the decrease of blood parameters (Hb: 6.9 mg/dL and Hct: 20%). A total of three units of red blood cell were transfused after the first day. Factor treatment was rearranged by hematologist at post-op fifth day. The blood parameters stabilized with APTT: 43.60 sec (29,5–40,8 reference interval) and fibrinogen: 594 mg/dL (200–400 mg/dL reference interval). The patient was discharged from the hospital at the postoperative day 12; factor VIII level was 64.6%; hemoglobin level was 11.1 g/dL.

Pin track infection was seen at the follow-up visits. It was treated with antibiotic therapy and aggressive dressing. The initial external fixator program was 0.25 mm/day compression at the nonunion site and 4 × 0.25 mm/day distraction at the second osteotomy site. Compression at nonunion side and distraction at distal part for lengthening were applied routinely until reaching the goal. Because the knee ankylosis limb length equalization is not achieved and four cm of shortening is accepted according to the decision of the patient. Union was fully seen on the X-ray under factor therapy. At the postoperative fifth month, dynamization of the system was done by removing the screws in stages to achieve rapid progress for consolidation. Union was fully seen on the X-ray at postoperative fourteenth month and the fixator was removed under local anesthesia (Figure 2).

**Figure 3:**
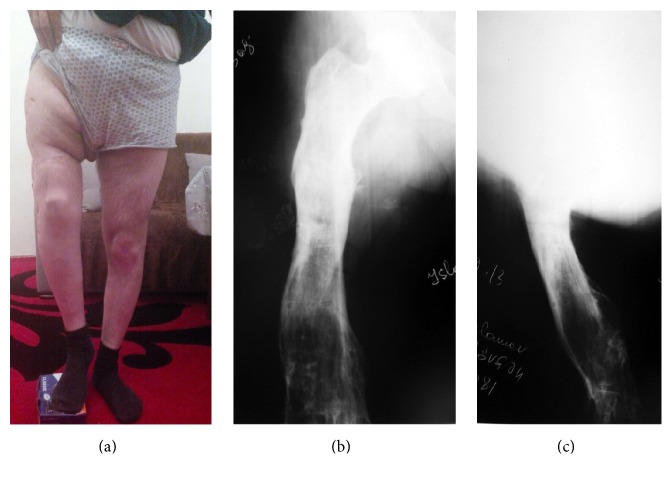
The patient was free of osteomyelitis and was able to walk with 4 cm shortness in the right lower extremity 7 years postoperatively. (a) Clinical appearance. (b, c) AP and lateral X-rays of the right femur seven years postoperatively.

Patient was followed up for 7 years. After this treatment, the patient was able to walk with weight bearing on the affected extremity with 4 cm shortening. The patient was free of osteomyelitis at the end of the seven years.  Figure 3 shows clinical appearance and X-rays at postoperative 7 years.

## 3. Discussion

As there are new factor prophylaxis regimens for hemophiliacs, we came across bleeding complications less frequently. On the other hand, as life expectancy increases, long-term complications must be overcame, such as fracture, nonunion, deformity, and osteomyelitis. Orthopedic surgeons have been performing many orthopedic procedures for hemophilia patients, such as fracture fixation, total joint arthroplasties, synovectomy, contracture release, and arthrodesis [1–3]. During this procedure, patients with hemophilia should be evaluated preoperatively, perioperatively, and postoperatively. The patients should have good understanding of the hemophilia, the surgery, and the required physiotherapy following the surgical procedure. Outcomes should also be discussed. Laboratory studies must be completed prior to a surgery especially inhibitor testing, prothrombin time, hepatitis C, and HIV. Factor replacement is the main step for preparing the surgery. It is recommended that the factor level should be corrected to 120% being close to the induction of anesthesia [4]. In this case, we followed the recommendation of our hematologist for factor replacement; it was similar to the literature.

External fixators generally use for treatment fixed flexion contracture and arthrodesis in hemophilia population. This study shows that the lengthening of the long bones by the external fixators is also effective and safe method to be used in hemophilia patients.

Distraction osteogenesis has been shown to be a reliable method for reconstructing segmental femur defects [5]. Fixator applications are generally used for the correction of flexion contracture of the knee or joint distraction for hemophiliac patients in the literature [6, 7]. The use of this technique has been applied to a wide range of orthopedic problems caused by such diverse etiologies as congenital disease, metabolic conditions [8], infections [5], traumatic injuries, nonunion [9], and congenital short stature [10, 11]. In this case, we used unilateral external fixator to obtain distraction osteogenesis, which could be safe with appropriate factor replacement for hemophiliac patients who have limb length discrepancy, nonunion, and osteomyelitis. In addition, this is the first report in the literature in which distraction osteogenesis was performed on the patient with hemophilia A. At the end of the treatment, we were able to achieve 6 cm femoral lengthening and treatment of the nonunion of the femur with the unilateral external fixator in hemophilia A patient.

Although radical debridement, acute compression, and lengthening are recommended for nonunion with bone defects due to osteomyelitis in the literature [12, 13], we preferred additionally bone grafting to treat nonunion and shortening for this extreme hemophilia patient because of the lack of biological factors, such as avascularity of the bone end atrophic pseudarthrosis formation. In addition, the patient has no knee flexion; this can affect the union because of the long force arm.

## 4. Conclusion

By the invention of the factor concentrate, the surgical procedures have been successfully performed in patients with hemophilia. To increase the rate of a successful outcome and decrease the rate of complications, these procedures should be performed in hospitals where there is an established center with all the specialists available. Limb lengthening and treatment of nonunion with the external fixators could be applicable and successful surgical procedures for the hemophilia patient.

## References

[1] Rodríguez-Merchán E. C. (2012). Orthopedic surgery is possible in hemophilic patients with inhibitors. *The American Journal of Orthopedics*.

[2] Zülfikar B., Akgül T., Özdemir N., Bezgal F., Talu U. (2013). Successful management of bilateral total hip replacement in a patient with von Willebrand's disease and developmental hip dysplasia. *Haemophilia*.

[3] Rodriguez-Merchan E. C. (2002). Orthopaedic surgery of haemophilia in the 21st century: an overview. *Haemophilia*.

[4] Wiedel J., Stabler S., Geraghty S., Funk S. (2010). Joint replacement surgery in hemophilia.

[5] Kocaoglu M., Eralp L., Ur Rashid H., Sen C., Bilsel K. (2006). Reconstruction of segmental bone defects due to chronic osteomyelitis with use of an external fixator and an intramedullary nail. *The Journal of Bone & Joint Surgery—American Volume*.

[6] Balci H. I., Kocaoglu M., Eralp L., Bilen F. E. (2014). Knee flexion contracture in haemophilia: treatment with circular external fixator. *Haemophilia*.

[7] Van Meegeren M. E. R., Van Veghel K., De Kleijn P. (2012). Joint distraction results in clinical and structural improvement of haemophilic ankle arthropathy: a series of three cases. *Haemophilia*.

[8] Kocaoglu M., Bilen F. E., Sen C., Eralp L., Balci H. I. (2011). Combined technique for the correction of lower-limb deformities resulting from metabolic bone disease. *The Journal of Bone & Joint Surgery—British Volume*.

[9] Arora S., Batra S., Gupta V., Goyal A. (2012). Distraction osteogenesis using a monolateral external fixator for infected non-union of the femur with bone loss. *Journal of Orthopaedic Surgery*.

[10] Sangkaew C. (2008). Distraction osteogenesis of the femur using conventional monolateral external fixator. *Archives of Orthopaedic and Trauma Surgery*.

[11] Park K.-W., Garcia R.-A. N., Rejuso C. A., Choi J.-W., Song H.-R. (2015). Limb lengthening in patients with achondroplasia. *Yonsei Medical Journal*.

[12] Liu T., Zhang X., Li Z., Peng D. (2011). Management of combined bone defect and limb-length discrepancy after tibial chronic osteomyelitis. *Orthopedics*.

[13] Yin P., Ji Q., Li T. (2015). A systematic review and meta-analysis of ilizarov methods in the treatment of infected nonunion of tibia and femur. *PLoS ONE*.

